# The Effectiveness of *Crataegus orientalis* M Bieber. (Hawthorn) Extract Administration in Preventing Alveolar Bone Loss in Rats with Experimental Periodontitis

**DOI:** 10.1371/journal.pone.0128134

**Published:** 2015-06-01

**Authors:** Mükerrem Hatipoğlu, Mehmet Sağlam, Serhat Köseoğlu, Ekrem Köksal, Ali Keleş, Hacı Hasan Esen

**Affiliations:** 1 Department of Periodontology, School of Dentistry, Akdeniz University, Antalya, Turkey; 2 Department of Periodontology, School of Dentistry, Izmir Katip Celebi University, Izmir, Turkey; 3 Department of Chemistry, Faculty of Sciences and Arts, Erzincan University, Erzincan, Turkey; 4 Department of Endodontics, School of Dentistry, Ondokuz Mayıs University, Samsun, Turkey; 5 Department of Pathology, School of Medicine, Necmettin Erbakan University, Konya, Turkey; UAMS, UNITED STATES

## Abstract

The purpose of this animal study was to evaluate the effects of hawthorn (*Crataeus orientalis* M Bieber.) extract on serum oxidative status and alveolar bone loss in experimental periodontitis. Twenty-seven Wistar rats were assigned to one of the following groups: non- ligated+placebo (saline) (NL, n = 9), ligature only+placebo (saline) (LO, n = 9), and ligature and treated with hawthorn extract in saline (H, n = 9) (100 mg/kg orogastrically, once a day for 11 days). Periodontitis was induced by submerging a 4/0 silk ligature in the sulcus of the mandibular right first molars of rats, and the animals were sacrificed after 11 days. Micro-CT examinations were performed for linear and volumetric parameter assessment of alveolar bone. Periodontal tissues were histopathologically examined to assess the differences among the study groups. Levels of serum total antioxidant status (TAS)/total oxidant status (TOS), and oxidative stress index (OSI) were also analyzed. Alveolar bone loss was significantly reduced by hawthorn administration compared to LO group (p<0.05). The number of inflammatory cells and osteoclasts in the LO group was significantly higher than that of the NL and H groups (p< 0.05). The number of osteoblasts in the LO and H groups was significantly higher than that of the NL group (p<0.05). TOS and OSI levels were significantly reduced in H group compared to LO group (P <0.05) and TAS levels were similar in H and NL group (p< 0.05). Hawthorn extract showed inhibitory effect on periodontal inflammation and alveolar bone loss by regulating TAS, TOS and OSI levels in periodontal disease in rats when administered systemically.

## Introduction

Periodontitis is a disease of oral cavity and the primary clinical features of periodontitis include gingival inflammation, periodontal pocketing, alveolar bone loss and clinical attachment loss [1]. This disease occurs by an interaction between dental microbial plaque and host response. The microbial challenge causes an increased host response in the periodontium that is characterized by the extreme production of inflammatory cytokines (e.g.interleukins, tumor necrosis factor-α), enzymes [including the matrix metalloproteinases (MMPs)] and prostanoids (e.g. prostaglandin E2) [2]. These pro-inflammatory mediators are responsible for the majority of periodontal destruction. Excessive production of reactive oxygen species (ROS) by polymorphonuclear leukocytes is one of the important pathologic conditions in the periodontitis, and it causes periodontal tissue damage through oxidizing DNA, proteins, and lipids [3, 4]. It was reported that ROS are able to induce destruction of periodontal tissues and are related to osteoclastic bone resorption [5].

Hawthorn (*Crataegus spp*.) belongs to the Rosaceae family and grows in northern mild regions such as Europe, East Asia, and eastern North America [6]. It has been reported that the genus *Crataegus* contains nearly 21 species in Turkey [7]. *Crataegus orientalis* M Bieber. (also known as Anatolian hawthorn) is indigenous to the Mediterranean region, Crimea, Turkey, and western Iran [8]. The prepared extracts or tinctures from hawthorn leaves, flowers, and/or fruits have been used traditionally since ancient times [9]. The plant is commonly used in the treatment of cardiovascular diseases [10]. Additionally, considering the pharmacological studies, hawthorn extracts may also be used as antiinflammatory and antioxidant agents [8]. It has been reported that hawthorns fruits have high flavonoid, vitamin C, glycoside, anthocynaidin, saponin, tannin, and antioxidant levels [10]. In another study, it was demonstrated that the water and ethanol extracts of hawthorn had H_2_O_2_ radical scavenging and total antioxidant activity when compared to standards such as butylated hydroxyanisole and α-tocopherol [11]. In a review, it was indicated that oral administration of extract provided a dose-dependent effect in a model of carrageenan-induced rat paw edema and showed anti-inflammatory activity [12]. Some researchers also demonstrated that hawthorn extract has bactericidal effects [13, 14]. Niu et al. reported that the antibacterial effect of ethanol extract was better than water extract against *Staphylococcus aureus* and *Klebsiella pneumonia* [15]. Flavonoids and oligomeric proanthocyanidins (OPCs), the fundamantal components of hawthorn, play role in most of the pharmacological activity [6].

Based on the antiinflammatory and antioxidant properties of hawthorn, we hypothesized that it may be beneficial in suppressing periodontal inflammation and alveolar bone loss in periodontal disease. Therefore, the present study investigates the effects of systemic hawthorn administration on serum oxidative stress and alveolar bone loss in experimental periodontitis model in rats.

## Materials and Methods

### Experimental Design

All animal experiment procedures in this study were approved by the Animal Ethics Committee of Akdeniz University School of Medicine (Permit Number:98/2014). Twenty-seven male Wistar rats (weight: 340±10g, 4 mo of age) were divided into three groups of nine animals each: non-ligated+placebo (saline) (NL), ligature only+placebo (saline) (LO), and ligature and treated with hawthorn extract in saline (H) (100 mg/kg per day for 11 days). They were housed in specially designed wire cages and maintained on a 12 h–12 h light–dark cycle with a constant room temperature of 25°C.

### Preparation of Hawthorn Extract

The ripe fruits of hawthorn were collected from a spice store from Erzincan, Turkey. The identification of hawthorn was performed by an an expert botanist. Mature fruit is orange or various shades of red, almost round in shape. The fruits of hawthorn were dried at 25°C and powdered in a grinder. The powder was extracted using ethanol (70%) and after filtration (sterile gauze), was concentrated using a vacuum evaporator at 40°C, under reduced pressure. Pharmacologic assays were carried out using dry crude extract dissolved in saline solution (vehicle).

### Experimental Periodontal Disease Induction and Administration of Hawthorn Extract

Experimental periodontitis induction was performed as in our previous animal studies [16, 17]. Animals were anesthetized with ketamine (40 mg/kg) and experimental periodontitis was induced by placing a 4/0 silk suture in a subgingival position around the right mandibular first molars. The ligatures were controlled every day, displaced apically into the gingival sulcus to make sure a subgingival position. The animals were housed in individual wire cages and received water and standard rat chow pellets ad libitum in both preligation and postligation periods.

In the test group, the rats received hawthorn in saline orogastrically, at a dosage of 100 mg/kg/day at placement of the ligature and daily until sacrifice. The ligatures were kept subgingivally for 11 days. Gingival inflammation signs (e.g. erythema and swelling) were noticed in the gingival tissues around the ligatured teeth. There was no animal death at the end of the study. After 11 days blood samples were obtained, the rats were sacrificed and their mandibles were split in two equal pieces from the midline between the incisor teeth after surgical dissection. The right piece of the mandibles were placed in bottles contains 10% formalin as in our previous study [17]. The blood samples were centrifuged at 3000x g for 10 min to obtain serum. Subsequently, the serum samples were collected into a sterile polyproplene tube and kept at -80°C until being analysed for the total antioxidant status (TAS), total oxidant status (TOS), and oxidative stress index (OSI).

### Microcomputed tomography (micro-CT) imaging

All mandibular samples were scanned using a high-resolution CT system (SkyScan 1172; Bruker-microCT, Kontich, Belgium). The X-ray tube was operated at 100 kV and 100 μA using a 0.5 mm Al+Cu filter with a resolution of 7.93 μm pixels. Scanning was performed by 180° rotation around the vertical axis, camera exposure time of 750 ms, rotation step of 0.3°, frame averaging of 4, and random movement of 20. Each specimen was scanned for a total of 60 min. The resulting two dimensional images (8-bit TIFF) were used to reconstruct axial cross-sections. Axial cross sections of the samples were reconstructed using the system-reconstruction software (NRecon v.1.6.3, SkyScan, Brucker-microCT) with a beam hardening correction of 55%, smoothing of 2, and an attenuation co-efficient range of 0–0.0720. Three-dimensional reconstructions were performed by using the same system-reconstruction software (NRecon v.1.6.3, SkyScan, Brucker-microCT). CTAn v.1.12 (Skyscan, Bruker-microCT) and CT Vol v.2.2.1 (Skyscan, Bruker-microCT) softwares were used for linear and volumetric analysis.

### Linear and Volumetric Alveolar Bone Measurements

#### Linear measurements

**Fig 1:**
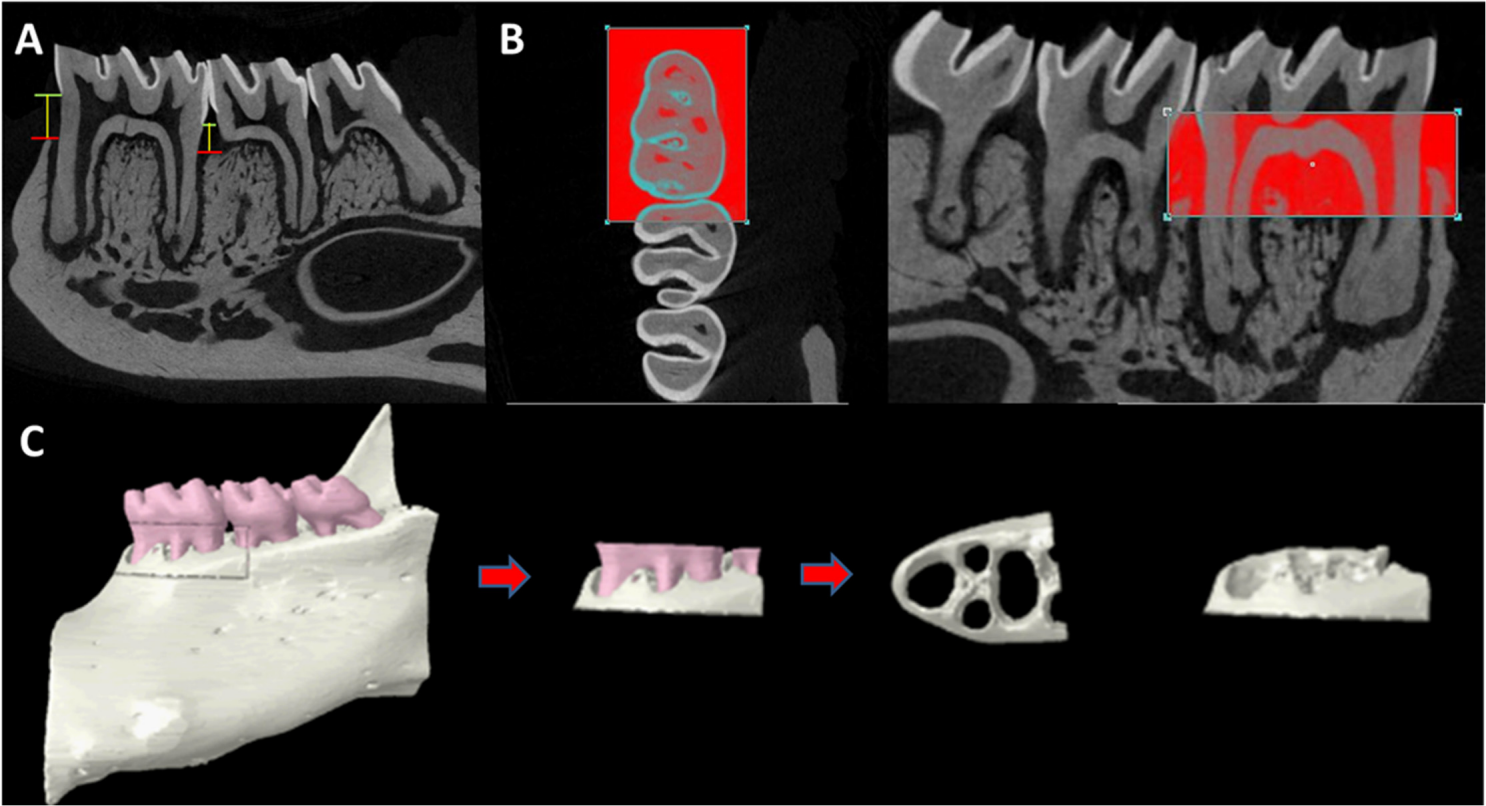
A- Linear measurements are taken of ABL in the interdental space from the CEJ to ABC. (Green line: CEJ, red line: ABC, yellow line: Distance (mm)). **B-** Axially, a rectangular-shaped region of interest that is 0.33 mm far from each direction of tooth crown. Vertically, a rectangular-shaped region of interest include coronal halves of the slices through mesial root apex to the CEJ. C- Volumetric measurement with 3-D generated ROI.

The alveolar bone loss (ABL) was assessed from images of two-dimensional (2D) micro-CT sections. Linear measurements were taken (in mm) from the cemento-enamel junction (CEJ) to the alveolar bone crest (ABC) at the distal and mesial root of the first molar. All images were reoriented so that both the CEJ and the root apex appeared in the same micro-CT slice (Fig 1A).

#### Volumetric micro-CT measurements

As each mandibular first molar is different in size, a standard region of interest (ROI) has been formed according to the size of the first molar, instead of forming a fixed ROI for each sample. Axially, a rectangle that is 0.33 mm far from each direction of tooth crown has been set as ROI (Fig 1B). Vertically, coronal halves of the slices through mesial root apex to the CEJ have been included to volume of interest (VOI) (Fig 1B). Thus, a standard VOI suitable for tooth size has been formed for each first molar. Tooth parts were removed from VOI and remaining bone volume in VOI has been recorded as mm^3^ (Fig 1C). Linear and volumetric analyses were performed by co-author (AK).

### Histopathologic Evaluation

The specimens were decalcified in 10% EDTA for 2 months and embedded in paraffin. Mesio-distally serial sections (5 μm) were taken and stained by using hematoxylin and eosin solutions. Two sections (5μm) at x200 magnification were used for light microscopy assessment. The interdental area between first and second molars was selected for histopathologic evaluation. The number of osteoblasts, osteoclasts, and inflammatory cell infiltrate (ICI) were analyzed in connective tissue, periodontal ligament and interdental septum by co-author (HHE). All of the osteoblasts (mononuclei, cuboidal cells around the active bone formation surfaces) and osteoclasts (multiple nuclei, ruffled border, and granular cytoplasm) were counted in the periodontium morphologically as in our previous studies [16, 17]. TRAP staining (Sigma Chemical Co., St Louis, MO, USA) was performed to identify and quantify osteoclasts.

### Evaluation of TAS, TOS, and OSI Levels

TAS, TOS and OSI levels were determined as in our previous study [17]. Serum TAS was analysed by using a commercially available kit (Fully Automated 3rd Generation Total Antioxidant Status (TAS) ASSAY KIT Product Code: RL0017, REL Assay Diagnostics, Mega Tıp, Gaziantep, Turkey) developed by Erel [18] In this assay, the antioxidative effect of the sample against a potent free-radical reaction initiated by the hydroxyl radical produced, is measured. The results are obtained as millimoles of trolox equivalent per liter (mmol trolox equiv/l). Serum TOS was analysed by using a commercially available kit (Fully Automated Total Oxidant Status (TOS) ASSAY KIT Product Code: RL0024, REL Assay Diagnostics, Mega Tıp) developed by Erel [18] The assay is calibrated with hydrogen peroxide, and the results are obtained in micromolar hydrogen peroxide equivalent per liter (μmol H_2_O_2_ equiv/l). TAS and TOS levels were analysed by co-author (SK).OSI is an indicator of the degree of oxidative stress. The percentage ratio of the TOS to the TAS is used to calculate OSI. To perform the calculation, the result unit of TAS, millimole of Trolox equivalent per liter, was converted to micromole equivalent per liter, and the OSI value was calculated by the formula OSI = [(TOS, μmol/L)/(TAS, μmol Trolox equivalent/L) × 100].

### Statistical analysis

The statistical analysis was performed using a commercially available software program (SPSS 20.0, IBM, Chicago, IL, USA). The Shapiro-Wilk normality test was used to verify the normality of the data. The data were normally distributed and parametric tests were used. ANOVA test followed by Tukey’s test for pair-wise comparisons were used for analyzing the number of osteoclast, osteoblast, inflammatory cells, alveolar bone loss (linear and volumetric measurements), TAS, TOS and OSI values. A value of p<0.05 was considered statistically significant.

## Results

**Fig 2:**
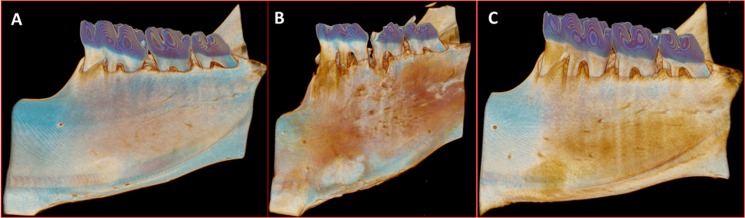
The 3D representative images of each group. A- NL group. B- LO group. C- H group.

Any signs of systemic illness were not observed throughout the study period. The 3D images representive of each group are shown in Fig 2.

### Micro-CT Findings

**Fig 3:**
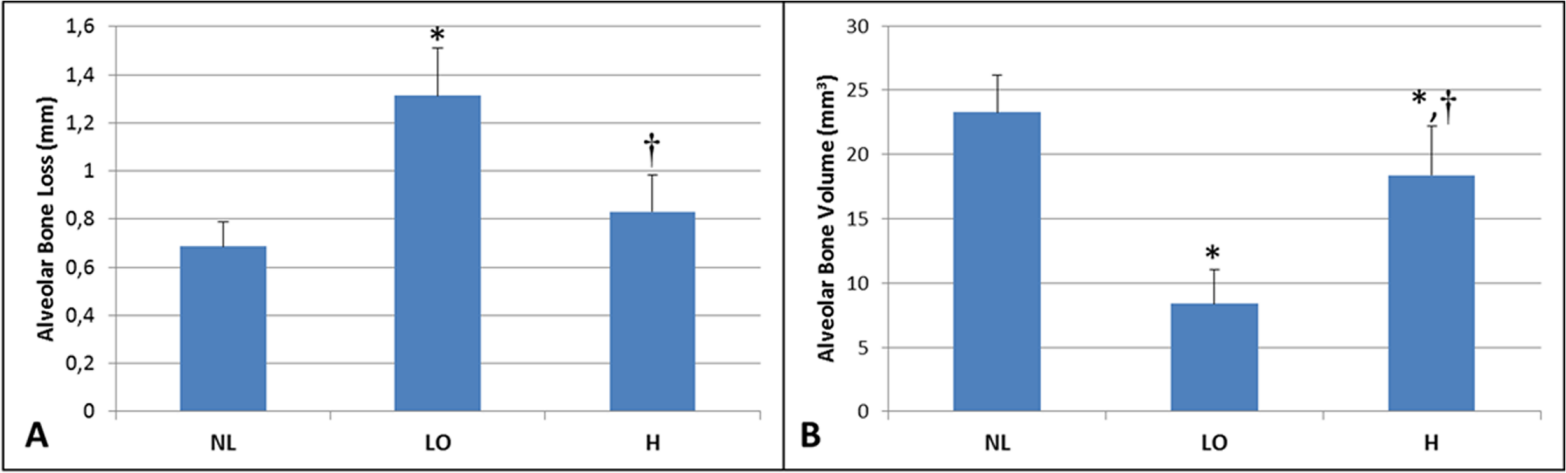
A- Mean ABL in study groups (mm). **B**- Alveolar bone volume in study groups (mm^3^). * Significant difference compared to NL group. † Significant difference compared to LO.

Significantly greater alveolar bone loss (mm) was observed in the LO group (1.31±0.19) compared to H (0.83±0.14) and NL (0.68±0.10) groups (P<0.05; Fig 3A). The alveolar bone loss was similar in the H and NL groups (P>0.05). Alveolar bone volume (mm^3^) was lower in the LO group (8.37±2.63) compared to H (18.38±3.81) and NL (23.27±2.90) groups (P<0.05).There was a significant difference between NL and H groups (P<0.05; Fig 3B).

### Histopathologic Findings

**Fig 4:**
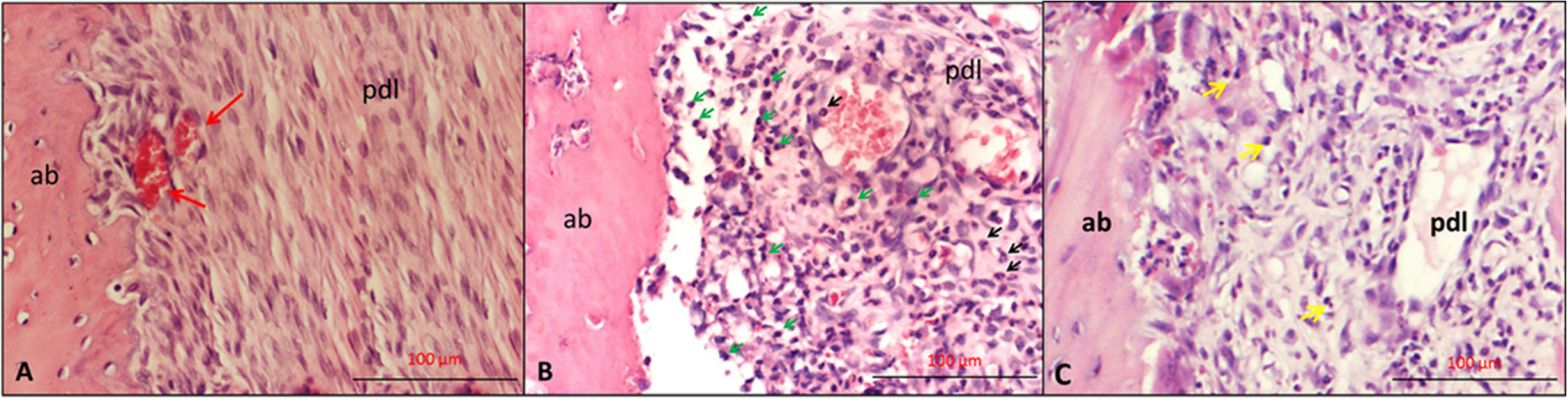
The histological representive images of each experimental group. A- The histological images of NL group showing normal periodontium (red arrows: erythrocytes). **B**- LO group showing intense inflammatory cells (green arrow: lymphocyte, black arrow: neutrophils) and dilated blood vessels **C-** The histological images of H group showing moderate inflammatory cell infiltrate in periodontal ligament (Yellow arrows show inflammatory cells). (ab: alveolar bone, pdl: periodontal ligament).

**Fig 5:**
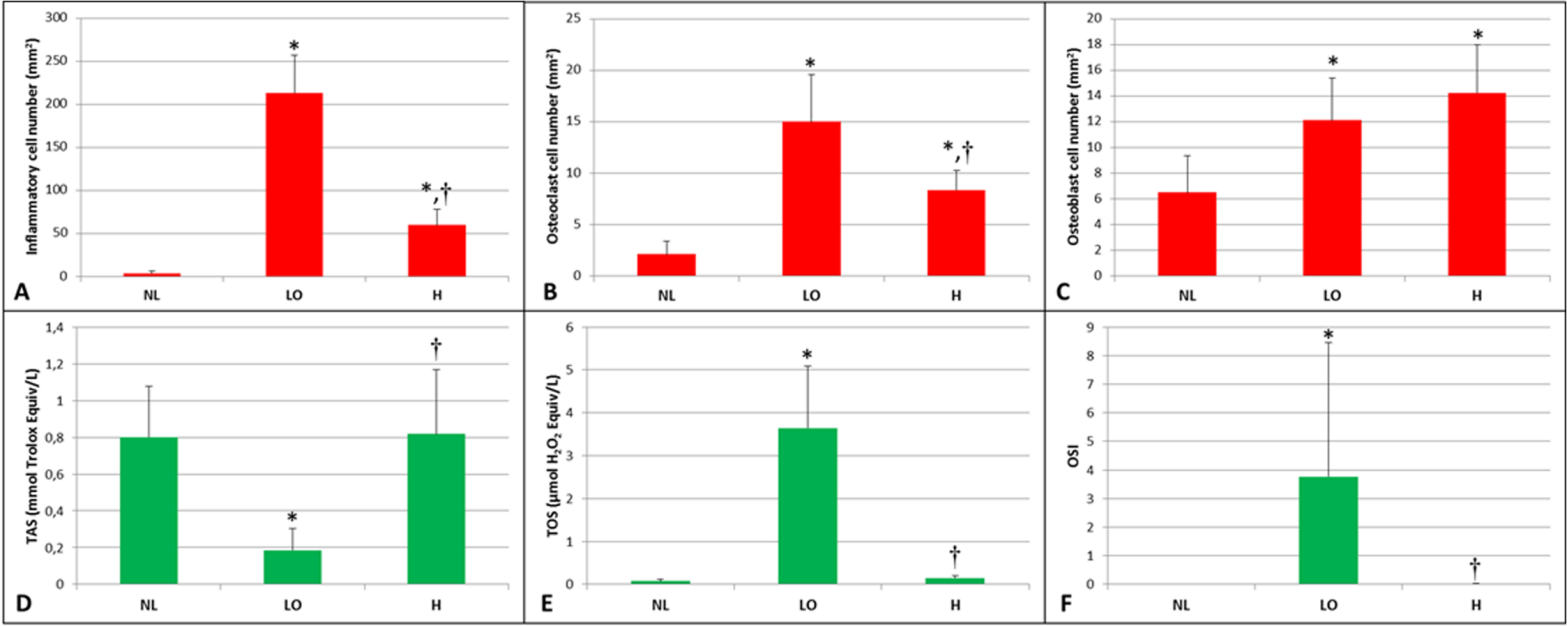
A- The number of inflammatory cells (/mm^2^) in the study groups. **B-** The number of osteoclasts (/mm^2^) in the study groups. **C-** The number of osteoblasts (/mm^2^) in the study groups. **D-** TAS in the study groups. **E-** TOS in the study groups. **F-** OSI levels in the study groups. * Significant difference compared to NL group. † Significant difference compared to LO.

The histological images representative of each experimental group are shown in Fig 4. The inflammatory infiltrate (/mm^2^) numbers in the study groups were presented in Fig 5A. The number of inflammatory infiltrate was greater in the LO group (213.56±43.32) compared with the H (59.87±18.62) and NL (4.12±2.58) groups (p<0.05). There was also significant difference between H and NL groups (p<0.05).

Fig 5B presents number of osteoclasts (/mm^2^) in the study groups. Significantly higher numbers of osteoclasts were observed in the LO group (15±4.56) compared with H (8.37±1.92) and NL (2.12±1.24) groups (p<0.05). Significant difference was also found between H and NL groups (p<0.05).

The number of osteoblasts (/mm^2^) was presented in Fig 5C for the study groups. Osteoblasts were found in all rats. The osteoblasts numbers were greater in the LO (12.12±3.27) and H (14.25±3.73) groups compared with NL group (6.5±2.87) (p<0.05). The osteoblast numbers were similar in LO and H groups (p>0.05).

### Serum TAS, TOS and OSI Levels

TAS, TOS and OSI levels were presented for each group in Fig 5. TAS levels were significantly lower in LO group (0.18±0.11) compared to NL (0.80±0.27) and H (0.81±0.35) groups (p<0.05; Fig 5D). TAS levels were similar in NL and H groups (p>0.05; Fig 5D). TOS levels were significantly higher in LO group (3.64±1.45) compared to NL (0.07±0.04) and H (0.14±0.05) groups (p<0.05; Fig 5E). TOS levels were similar in NL and H groups (p>0.05; Fig 5E). OSI levels were significantly higher in LO group (3.76±4.69) compared to NL (0.01±0) and H (0.02±0.02) groups (p<0.05; Fig 5F). OSI levels were similar in NL and H groups (p>0.05; Fig 5F)

## Discussion

Hawthorn species are medicinal plants used as a folk medicine, which have flavonoids, triterpene acids, proanthocyanidins, and organic acids as essential components. Cardiovascular protective activity of hawthorn fruit has come into prominence in numerous pharmacological studies. Additionally, some studies have reported on antioxidant, antiinflammatory and anticarcinogenic effects of hawthorn [13].

To our knowledge, this is the first study evaluating the effect of hawthorn extract administration on serum oxidative status and alveolar bone loss in experimental periodontitis model in rats.

Animal models for periodontitis have both advantages and disadvantages. Various animals are used for periodontitis model such as monkeys, dogs, pigs and rats [19, 20]. Although monkeys, pigs and dogs develop natural or experimental periodontitis similar to humans, the expense of, special husbandry requirements and ethical issues for these animals limit their use in periodontal studies [19, 20]. Rats are resistant to the occurrence of periodontitis but they are easy to handle and used in experimental periodontitis models because periodontal anatomy is quite similar to that observed in humans [19, 20]. Also they can be obtained with different microbial status and genomes [20]. Humans are not equally susceptible to the periodontal infections, but rats used in experimental periodontitis models have similar susceptibility to periodontal disease [19].

Various techniques have been described in the literature to induce periodontitis in rats, such as the introduction of pathogenic microorganisms, dietary manipulation and the ligature placement [21–23]. Lima et al [24] reported that inflammatory cells, involving lymphocytes and osteoclasts, appeared underneath the ligature; significant alveolar bone loss was induced after 7 days, reached a peak point between days 7 and 11, and declined at day 14. In another study, Lima et al [25] indicated that remarkable inflammatory cell infiltration, and intense cementum destruction were shown on the 11th day after periodontitis induction. Furthermore, they also reported that leukocytosis with significant neutrophilia at 6 hours and lymphomonocytosis at 11 days was observed, suggesting a systemic impact of the experimental periodontitis. In this present study, the ligature was placed around the first molar teeth for 11 days, as in previous studies [5, 26, 27].

In healthy condition, there is a balance among ROS and antioxidants [28]. A disturbance in favour of ROS production causes oxidative stress under pathological conditions [29]. Excessive ROS production has been associated with pathogenesis of many human diseases such as atherosclerosis, diabetes mellitus and periodontal diseases [28, 29]. Many researches have shown the antioxidant effect of Hawthorn. It was demonstrated that the genotoxicity induced by gamma irradiation was reduced through antioxidant activity of hawthorn extract in bone marrow cells [30]. Popovic-Milenkovic et al. reported that Species *Crataegus nigra* fruits hydroalcoholic extract showed antioxidant activity [31]. In another study, it was indicated that flowers, leaves and fruits of *C*. *monogyna* had H_2_O_2_ radical scavenging, total antioxidant activity and may be used as source of natural antioxidants [11]. Bor et al. demonstrated that ethanol extract of hawthorn exhibits noticeable antioxidant activity *in vivo*[8] TOS and OSI are indicators of the degree of oxidative stress [32]. In our study, it was observed that serum TOS and OSI levels were higher in LO group compared to the NL group. Systemic Hawthorn administration significantly reduced TOS and OSI levels compared to LO group. While TAS levels were similar in NL and H groups, higher in H group compared to LO group. This condition may be explained with antioxidant effect of hawthorn extract. Increased TAS and decreased TOS and OSI levels in H group may reduce alveolar bone loss compared to LO group in this present study.

Although osteoblast numbers were similar in H and LO groups, the lower osteoclast number in H group compared to LO group were observed in our study. In various *in vitro* and *in vivo* studies, it was indicated that oxidative stress diminished the level of bone formation by depressing the osteoblasts differentiation and survival [33]. Additionally, it has been demonstrated that ROS stimulate bone resorption through activating osteoclasts [33]. Decreased osteoclast number in H group may be explained by inhibitory effect of hawthorn on oxidative stress based on TOS and OSI levels in our study. Various studies demonstrated that antioxidant agents provide increased osteoblast and decreased osteoclast numbers in experimental rat periodontitis models [16, 17, 26]. This present study was in agreement with these studies.

In the literature, anti-inflammatory effect of hawthorn has been also demonstrated. Kao et al. reported that the release of PGE2 and nitric oxide as induced by lipopolysaccharide were decreased by the dried fruits of *Crataegus pinnatifida* in macrophage RAW 264.7 cells. In the same study they also observed that *Crataegus pinnatifida* decreased the hepatic expression of iNOS and COX-2 induced by LPS in rats [34]. In another study, it was demonstrated that *C*. *orientalis* significantly inhibited carrageenan-induced paw edema in mice and displayed a dose-dependent antiinflammatory effect [8]. Malekinejad et al. reported that hydroalcoholic extract of hawthorn berries decreased colitis-induced edema and infiltration of neutrophils in experimentally induced colitis in rats [35]. It was also known that ROSs play an important role in tissue damage by stimulation of pro-inflammatory cytokine release by monocytes and macrophages [36]. Considering this finding, hawthorn extract may show an anti-inflammatory effect because of its antioxidant activity. In our study, inflammatory cell infiltrate was significantly lower in H group compared to LO group. This observation may be related to anti-inflammatory effect of hawthorn.

Various dosages of hawthorn extract have been used in studies. Kao et al. used 50, 100 and 200 mg/kg hawthorn extract in rats to investigate the anti-inflammatory potential of the flavonoid contents from dried fruits of *C*. *pinnatifida in vivo* [34]. In another study, the same dosages in study of Kao et al [34] were used to evaluate the anti-inflammatory effect of hawthorn in mice. They demonstrated that the liver pathological changes caused by LPS in the animals pretreated with hawthorn (100 mg/kg) were reduced [8]. Malekinejad et al. used 100 mg/kg hawthorn extract to investigate the protective effect of hydroalcoholic extract of hawthorn berries on acetic acid–induced colitis in rats [35]. It was stated that mice and rats have been safely given a standardized extract at doses up to 3 g/kg body weight [12]. Unremarkable adverse events have been shown in studies using excessive dosing of hawthorn flower extract (600 mg/kg/day; flavonoids) over 30 days for rats [12]. We used 100 mg/kg hawthorn extract orogastrically as in previous study of Malekinejad which antioxidant and anti-inflammatory effects of hawthorn were observed [35]. We observed no adverse effects in animals.

Local applications has some advantages over the systemic administration, including the maintenance of significant concentrations of agent for sufficient lengths of time within the periodontal pocket and minimal or no side effects [37]. Local application could be more preferable if its antibacterial effect was evaluated in this present study. But we aimed to investigate the possible antioxidant and antiinflammatory effect of hawthorn. Thus we preferred the systemic route.

In conclusion, we observed that systemic administration of hawthorn extract has beneficial effects in reducing inflammation and alveolar bone loss by regulating TAS, TOS and OSI levels in periodontal disease in rats. Studies evaluating antibacterial effect of hawthorn extract should be conducted by deteminig the appropriate dosage with *in vitro* experiments in the future. Hawthorn extract may be clinically promising agent for the prophylaxis of periodontitis.

## References

[1] Flemmig TF. Periodontitis. Annals of periodontology / the American Academy of Periodontology. 1999;4(1):32–8. 10.1902/annals.1999.4.1.32 . 10863373

[2] Preshaw PM. Host response modulation in periodontics. Periodontology 2000. 2008;48:92–110. 10.1111/j.1600-0757.2008.00252.x . 18715360

[3] Chapple IL, Matthews JB. The role of reactive oxygen and antioxidant species in periodontal tissue destruction. Periodontology 2000. 2007;43:160–232. 10.1111/j.1600-0757.2006.00178.x . 17214840

[4] Luqman S, Rizvi SI. Protection of lipid peroxidation and carbonyl formation in proteins by capsaicin in human erythrocytes subjected to oxidative stress. Phytotherapy research: PTR. 2006;20(4):303–6. 10.1002/ptr.1861 . 16557614

[5] Toker H, Ozdemir H, Eren K, Ozer H, Sahin G. N-acetylcysteine, a thiol antioxidant, decreases alveolar bone loss in experimental periodontitis in rats. Journal of periodontology. 2009;80(4):672–8. 10.1902/jop.2009.080509 . 19335088

[6] Yao M, Ritchie HE, Brown-Woodman PD. A reproductive screening test of hawthorn. Journal of ethnopharmacology. 2008;118(1):127–32. 10.1016/j.jep.2008.03.020 . 18485639

[7] Sözer U, Dönmez A.A., Meriçli A.H. Constituents from the leaves of Crataegus davisii Browicz. Sci Pharm. 2006; 74:203–8.

[8] Bor Z, Arslan R, Bektaş N., Pirildar S., Dönmez A.A. Antinociceptive, antiinflammatory, and antioxidant activities of the ethanol extract of Crataegus orientalis leaves. Turk J Med Sci. 2012;42 (2):315–24.

[9] Bahorun T, Aumjaud E, Ramphul H, Rycha M, Luximon-Ramma A, Trotin F, et al Phenolic constituents and antioxidant capacities of Crataegus monogyna (Hawthorn) callus extracts. Die Nahrung. 2003;47(3):191–8. 10.1002/food.200390045 . 12866623

[10] Ljubuncic P, Portnaya I, Cogan U, Azaizeh H, Bomzon A. Antioxidant activity of Crataegus aronia aqueous extract used in traditional Arab medicine in Israel. Journal of ethnopharmacology. 2005;101(1–3):153–61. 10.1016/j.jep.2005.04.024 . 15970411

[11] Keser S, Celik S, Turkoglu S, Yilmaz O & Turkoglu I. Hydrogen peroxide radical scavenging and total antioxidant activity of hawthorn Chemistry Journal 2012;2(01):9–12.

[12] Kumar D, Arya V, Bhat ZA, Khan NA, Prasad DN. The genus Crataegus: chemical and pharmacological perspectives. Rev Bras Farmacogn / Braz J Pharmacogn 2012:10.1590/S0102-695X2012005000094

[13] Tadic VM, Dobric S, Markovic GM, Dordevic SM, Arsic IA, Menkovic NR, et al Anti-inflammatory, gastroprotective, free-radical-scavenging, and antimicrobial activities of hawthorn berries ethanol extract. Journal of agricultural and food chemistry. 2008;56(17):7700–9. 10.1021/jf801668c . 18698794

[14] Benli M, Yigit N, Geven F, Guney K, Bingol U. Antimicrobial activity of endemic Crataegus tanacetifolia (Lam.) Pers and observation of the inhibition effect on bacterial cells. Cell biochemistry and function. 2008;26(8):844–51. 10.1002/cbf.1515 . 18946875

[15] Niu Y, Nan Y, Yuan L, Wang R. Study on antibacterial effect of medlar and hawthorn compound extract in vitro. African journal of traditional, complementary, and alternative medicines: AJTCAM / African Networks on Ethnomedicines. 2013;10(3):567–73. 24146490 10.4314/ajtcam.v10i3.27 PMC3777602

[16] Saglam M, Hatipoglu M, Koseoglu S, Esen HH, Kelebek S. Boric acid inhibits alveolar bone loss in rats by affecting RANKL and osteoprotegerin expression. Journal of periodontal research. 2014;49(4):472–9. 10.1111/jre.12126 . 24033134

[17] Saglam M, Koseoglu S, Hatipoglu M, Esen HH, Koksal E. Effect of sumac extract on serum oxidative status, RANKL/OPG system and alveolar bone loss in experimental periodontitis in rats. Journal of applied oral science: revista FOB. 2015;23(1):33–41. 10.1590/1678-775720140288 25760266 PMC4349117

[18] Erel O. A novel automated direct measurement method for total antioxidant capacity using a new generation, more stable ABTS radical cation. Clinical biochemistry. 2004;37(4):277–85. 10.1016/j.clinbiochem.2003.11.015 . 15003729

[19] Oz HS, Puleo DA. Animal models for periodontal disease. Journal of biomedicine & biotechnology. 2011;2011:754857 10.1155/2011/754857 21331345 PMC3038839

[20] Struillou X, Boutigny H, Soueidan A, Layrolle P. Experimental animal models in periodontology: a review. The open dentistry journal. 2010;4:37–47. 10.2174/1874210601004010037 20556202 PMC2885595

[21] Toker H, Ozan F, Ozer H, Ozdemir H, Eren K, Yeler H. A morphometric and histopathologic evaluation of the effects of propolis on alveolar bone loss in experimental periodontitis in rats. Journal of periodontology. 2008;79(6):1089–94. 10.1902/jop.2008.070462 . 18533788

[22] Robinson M, Hart D, Pigott GH. The effects of diet on the incidence of periodontitis in rats. Laboratory animals. 1991;25(3):247–53. . 1921324 10.1258/002367791780808374

[23] Buduneli E, Vardar S, Buduneli N, Berdeli AH, Turkoglu O, Baskesen A, et al Effects of combined systemic administration of low-dose doxycycline and alendronate on endotoxin-induced periodontitis in rats. Journal of periodontology. 2004;75(11):1516–23. 10.1902/jop.2004.75.11.1516 . 15633329

[24] de Lima V, Bezerra MM, de Menezes Alencar VB, Vidal FD, da Rocha FA, de Castro Brito GA, et al Effects of chlorpromazine on alveolar bone loss in experimental periodontal disease in rats. European journal of oral sciences. 2000;108(2):123–9. . 10768725 10.1034/j.1600-0722.2000.00766.x

[25] Lima V, Vidal FD, Rocha FA, Brito GA, Ribeiro RA. Effects of tumor necrosis factor-alpha inhibitors pentoxifylline and thalidomide on alveolar bone loss in short-term experimental periodontal disease in rats. Journal of periodontology. 2004;75(1):162–8. 10.1902/jop.2004.75.1.162 . 15025228

[26] Demirer S, Kara MI, Erciyas K, Ozdemir H, Ozer H, Ay S. Effects of boric acid on experimental periodontitis and alveolar bone loss in rats. Archives of oral biology. 2012;57(1):60–5. 10.1016/j.archoralbio.2011.07.012 . 21871607

[27] Ozdemir H, Kara MI, Erciyas K, Ozer H, Ay S. Preventive effects of thymoquinone in a rat periodontitis model: a morphometric and histopathological study. Journal of periodontal research. 2012;47(1):74–80. 10.1111/j.1600-0765.2011.01406.x . 21992581

[28] Bostanci V, Toker H, Senel S, Ozdemir H, Aydin H. Effect of chronic periodontitis on serum and gingival crevicular fluid oxidant and antioxidant status in patients with familial Mediterranean fever before and after periodontal treatment. Journal of periodontology. 2014;85(5):706–12. 10.1902/jop.2013.130230 . 23826647

[29] Baltacioglu E, Akalin FA, Alver A, Balaban F, Unsal M, Karabulut E. Total antioxidant capacity and superoxide dismutase activity levels in serum and gingival crevicular fluid in post-menopausal women with chronic periodontitis. Journal of clinical periodontology. 2006;33(6):385–92. 10.1111/j.1600-051X.2006.00923.x . 16677326

[30] Hosseinimehr SJ, Azadbakht M, Mousavi SM, Mahmoudzadeh A, Akhlaghpoor S. Radioprotective effects of hawthorn fruit extract against gamma irradiation in mouse bone marrow cells. Journal of radiation research. 2007;48(1):63–8. . 17185880 10.1269/jrr.06032

[31] Popovic-Milenkovic MT, Tomovic MT, Brankovic SR, Ljujic BT, Jankovic SM. Antioxidant and anxiolytic activities of Crataegus nigra Wald. et Kit. berries. Acta poloniae pharmaceutica. 2014;71(2):279–85. . 25272648

[32] Erel O. A new automated colorimetric method for measuring total oxidant status. Clinical biochemistry. 2005;38(12):1103–11. 10.1016/j.clinbiochem.2005.08.008 . 16214125

[33] Baek KH, Oh KW, Lee WY, Lee SS, Kim MK, Kwon HS, et al Association of oxidative stress with postmenopausal osteoporosis and the effects of hydrogen peroxide on osteoclast formation in human bone marrow cell cultures. Calcified tissue international. 2010;87(3):226–35. 10.1007/s00223-010-9393-9 . 20614110

[34] Kao ES, Wang CJ, Lin WL, Yin YF, Wang CP, Tseng TH. Anti-inflammatory potential of flavonoid contents from dried fruit of Crataegus pinnatifida in vitro and in vivo. Journal of agricultural and food chemistry. 2005;53(2):430–6. 10.1021/jf040231f . 15656684

[35] Malekinejad H, Shafie-Irannejad V, Hobbenaghi R, Tabatabaie SH, Moshtaghion SM. Comparative protective effect of hawthorn berry hydroalcoholic extract, atorvastatin, and mesalamine on experimentally induced colitis in rats. Journal of medicinal food. 2013;16(7):593–601. 10.1089/jmf.2012.2672 23875899 PMC3719480

[36] Chapple IL. Reactive oxygen species and antioxidants in inflammatory diseases. Journal of clinical periodontology. 1997;24(5):287–96. . 9178107 10.1111/j.1600-051x.1997.tb00760.x

[37] Greenstein G, Polson A. The role of local drug delivery in the management of periodontal diseases: a comprehensive review. Journal of periodontology. 1998;69(5):507–20. 10.1902/jop.1998.69.5.507 . 9623893

